# Studies on the Child Handbook in Brazil: a scoping review

**DOI:** 10.11606/s1518-8787.2023057004733

**Published:** 2023-07-31

**Authors:** Juliana Araujo Teixeira, Cintia de Freitas Oliveira, Maritsa Carla de Bortoli, Sonia Isoyama Venâncio

**Affiliations:** I Insper Centro Brasileiro de Pesquisa Aplicada à Primeira Infância São Paulo SP Brazil Insper. Centro Brasileiro de Pesquisa Aplicada à Primeira Infância. São Paulo, SP, Brazil; II Secretaria de Estado da Saúde Instituto de Saúde São Paulo SP Brazil Secretaria de Estado da Saúde. Instituto de Saúde. São Paulo, SP, Brazil

**Keywords:** Child Health, Growth and Development, Process Assessment, Health Care, Review

## Abstract

**OBJECTIVE:**

To systematically identify and map studies involving the *Caderneta da Criança* (Child Handbook - CH) in Brazil.

**METHODS:**

A scoping review using the methodology proposed by the Joanna Briggs Institute. The databases PubMed, *Biblioteca Virtual em Saúde* (BVS), *Biblioteca Digital Brasileira de Teses e Dissertações, Periódicos* Capes and Google Scholar were consulted. Primary and secondary studies that mentioned the use of CH were included, without restrictions regarding design, year of publication or population, published in Portuguese, English or Spanish and gray literature (theses and dissertations).

**RESULTS:**

A total of 129 studies were included, mostly descriptive, published as scientific papers. The Northeast region, the population of caregivers of children and the Primary Care scenario were observed in most studies; 47% of the studies used the CH as a source of data, the majority on vaccination. Despite the different criteria to define adequacy of completing the CH, the studies identified weaknesses in filling out all of its items, except for immunization. The utilization and/or completion of the CH were linked to various factors, including the availability of the CH, characteristics of the children (such as sex, age, prematurity and well-child appointments), attributes of the family members and caregivers (such as age, education, income, parity, work status, prenatal care, reading, note-taking and bringing CH to appointments) and features of the professionals (such as workplace, medical specialty, communication style, knowledge about the CH, requesting, guiding and note-taking).

**CONCLUSIONS:**

The results reinforce the need to better understand which factors affect the distribution of the CH in the population, as well as pointing to the need for understanding its use and completion by the different care points. The need for intervention studies to improve the implementation of this tool and to include training on the use of the CH in the context of continuing health education becomes evident.

## INTRODUCTION

The world’s progress in reducing child mortality is remarkable^1^. At this time, efforts are being directed not only to reduce mortality, but also to promote the full development of all children^2^. Thus, educational actions with families, monitoring and recording data on the child’s health, and the integration of actions between services are essential^3^.

Instruments for recording information, such as health records, have been used in more than 163 countries with the aim of improving maternal and child health^4,5^. A systematic review identified that health records for pregnant women and children are effective in improving health outcomes^6^. These records have a positive impact on promoting care-seeking behaviors, improving knowledge and care practices, encouraging home care for childhood illnesses, reducing child mortality and morbidity and facilitating ongoing care.

In Brazil, the Children’s Card was created in 1984 by the Ministry of Health (MoH), replacing the Vaccination Record, to include the monitoring of growth and child development (CD) of children aged 0 to 5 years^7,8^. This instrument underwent several updates, and in 2005, the Child Health Handbook (CHH) was created as a tool for comprehensive monitoring of child health. In 2021, the *Caderneta da Criança* (Child Handbook - CH) was launched, aimed at all children born in Brazilian territory, which provides for the monitoring of children aged 0 to 10 years. This new version was designed to be used by families and healthcare providers and other child care services, such as those in education and social assistance, facilitating the integration of actions^9,10^. The first part of the CH is directed at families, and the second part is for professional records for child monitoring, including a tool for autism screening^10^.

The CH is the main tool for monitoring healthy growth and development according to the National Policy of Comprehensive Healthcare for Children (PNAISC)^3^. Despite its potential, and being a nationwide action established by the MoH, studies have highlighted the inadequate use and completion of the CH, with no consensus regarding the factors associated with this use or interventions that can improve it^11,12^. Therefore, this review aims to map the studies on the MoH’s CH that have analyzed its use, barriers and facilitators, and interventions aimed at its use, in order to provide information for its effective implementation.

## METHODS

### Study Design

A scoping review was carried out, which is a systematic method for mapping the scientific production on a given topic with the aim of identifying concepts and research gaps^13^, using the methodology of the Joanna Briggs Institute^13^. The following question was considered, based on the PCC acronym (population, concept and context): What is the scientific production on the use of the CH by healthcare professionals, managers, family and caregivers in Brazil? The research protocol was published in the Zenodo repository (https://sandbox.zenodo.org/record/891923#.YQhK445KhPY).

### Eligibility Criteria

Primary and secondary studies that mentioned the use of the CH in the Brazilian context, regardless of study design, publication year, or population, published in Portuguese, English, or Spanish, were included. Grey literature such as theses and dissertations were included, while conference abstracts and undergraduate course papers were excluded. Studies that did not explicitly state that the instrument used referred to CH were only included if the study description left no doubt that it was the MoH instrument.

### Search and Selection of Studies

After mapping terms related to the research question and discussions among specialists, a search strategy was built for PubMed and subsequently adapted by two librarians for other research platforms. Supplementary Table 1^a^ shows the dates, terms, databases, and strategies used.

**Table 1:** Classification of studies according to the approach to the Child Handbook and type of study – CPAPI, 2022.

Approach to CH	Type of study	n	%	Citations
Data source	Article	50	75.8	18, 20, 26, 30, 31, 37, 39–43, 45, 47–49, 52, 55, 56, 58, 61, 64, 68–76, 85, 87, 100, 102, 105, 106, 109, 110, 114, 115, 118, 119, 121, 123, 124, 134, 142–145
	Review article	0	0	-
	Master’s dissertation	12	18.2	16, 21, 23, 24, 44, 50, 54, 57, 66, 67, 108, 111
	Doctoral thesis	4	6.1	32, 51, 101, 103
Object of study	Article	24	49	7, 17, 27, 33, 36, 53, 59, 63, 65, 77, 79–84, 86, 91, 92, 95, 112, 116, 130, 131
	Review article	6	12.2	132, 136–140
	Master’s dissertation	16	32.7	16, 22, 35, 60, 88, 90, 93, 96, 107, 122, 125, 127, 129, 133, 135, 141
	Doctoral thesis	3	6.1	34, 94, 99
Citation in results	Article	7	50	62, 78, 97, 98, 104, 126, 128
	Review article	2	14.3	11, 12
	Master’s dissertation	5	35.7	21, 24, 25, 113, 141
	Doctoral thesis	0	0	-
Data collection instrument	Article	4	57.1	19, 28, 29, 89
	Review article	0	0	-
	Master’s dissertation	3	42.9	23, 46, 57
	Doctoral thesis	0	0	-

Total of approaches to the Child Handbook: n = 136; article: n = 84 (65.1%); review article: n = 8 (6.2%); master’s dissertation: n = 30 (23.3%) and doctoral thesis: n = 7 (5.4%).

Screening of titles and abstracts and reading of full texts were performed by two independent reviewers using the Rayyan^14^platform, and disagreements were resolved by a third reviewer. In both stages, a sample of studies was selected at the beginning of the process for calibrating of the inclusion and exclusion criteria.

### Data Extraction

The data extraction process involved the development of a spreadsheet (Supplementary Table 2^b^) and three articles were selected for calibration. The studies were independently extracted by two reviewers and disagreements were resolved by a third. The following information was collected: (i) study characteristics, (ii) results, (iii) barriers and facilitators to the use of the CH, and (iv) limitations related to the CH. For each included study, the approach to the CH was identified: (i) data source (studies that collected information of interest to the authors about the child, through CH, for example, immunization data); (ii) object of study (the objective was to study the CH, for example, its completion and associated factors); (iii) citation in results (only cited CH in the results); and (iv) data collection instrument (used some CH instrument for data collection, such as the CD surveillance instrument).

**Table 2:** Characteristics of the studies according to their approach to the Child Handbook – CPAPI, 2022.

Variable	Total		Approach to CH studies							

			Data source		Object of study		Citation in results		Data collection instrument	
				
	n	%	n	%	n	%	n	%	n	%
									
	136	100.0	66	47.0	49	39.0	14	10.3	7	3.7
Nomenclature adopted by the MoH										
No	37	28.7	33	50.0	2	4.1	2	14.3	0	0.0
Yes	92	71.8	33	50.0	47	95.9	12	85.7	7	100.0
CH/MoH explicitly stated by the authors										
Not explicit	54	41.9	47	71.2	4	8.2	3	21.4	0	0.0
Explicit	75	58.1	19	28.8	45	91.8	11	78.6	7	100.0
Region of Brazil										
North	7	5.3	3	4.5	3	6.1	1	6.7	0	0.0
Northeast	57	42.8	30	44.8	20	40.8	7	46.7	6	66.7
Central-West	17	12.8	7	10.4	7	14.3	2	13.3	1	11.1
Southeast	41	30.8	21	31.3	15	30.6	4	26.7	2	22.2
South	11	8.3	6	9.0	4	8.2	1	6.7	0	0.0
Population										
Family members or caregivers	81	75.0	54	93.1	21	53.8	4	33.3	5	100.0
Healthcare professionals	17	15.7	1	1.7	12	30.8	6	50.0	0	0.0
Both	9	8.3	3	5.2	5	12.8	2	16.7	0	0.0
Others^a^	1	0.9	0	0.0	1	2.6	0	0.0	0	0.0
Scenario										
PHC	71	58.2	24	37.5	34	77.3	13	92.9	6	85.7
MAC	16	13.1	13	20.3	3	6.8	0	0.0	0	0.0
Community	16	13.1	15	23.4	1	2.3	0	0.0	0	0.0
Education	10	8.2	8	12.5	2	4.5	0	0.0	0	0.0
Social assistance	1	0.8	1	1.6	0	0.0	0	0.0	0	0.0
More than one scenario	8	6.6	3	4.7	4	9.1	1	7.1	1	14.3
Location										
UBS	38	32.5	14	22.2	14	33.3	8	66.7	6	85.7
Domicile	26	22.3	22	34.9	3	7.1	2	16.7	0	0.0
Vaccination campaign	8	6.8	2	3.2	5	11.9	1	8.3	0	0.0
Outpatient clinic	8	6.8	8	12.7	0	0.0	0	0.0	0	0.0
School	6	5.1	5	7.9	1	2.4	0	0.0	0	0.0
Hospital	5	4.3	4	6.3	1	2.4	0	0.0	0	0.0
More than one location	22	18.8	7	11.1	15	35.7	1	8.3	1	14.3
Others^b^	4	3.4	1	1.6	3	7.1	0	0.0	0	0.0
Investigated outcomes										
Completion of CH	44	43.1	-	-	37	44.0	9	45.0	-	-
Factors associated with CH completion	23	22.5	-	-	22	26.2	1	5.0	-	-
Healthcare professionals’ perceptions of CH	14	13.7	-	-	12	14.3	2	10.0	-	-
Implementation-related aspects	7	6.9	-	-	2	2.4	5	25.0	-	-
Healthcare professionals’ knowledge about CH	5	4.9	-	-	4	4.8	1	5.0	-	-
Factors associated with mother’s CH reading	2	2.0	-	-	2	2.4	0	0.0	-	-
Inspection of CH (vaccination) by healthcare professionals	2	2.0	-	-	1	1.2	1	5.0	-	-
Factors associated with CH use	1	1.0	-	-	1	1.2	0	0.0	-	-
Factors associated with bringing CH to appointments	1	1.0	-	-	1	1.2	0	0.0	-	-
Teaching about CH	1	1.0	-	-	1	1.2	0	0.0	-	-
Access to CH	1	1.0	-	-	0	0.0	1	5.0	-	-
Parents’ knowledge about CH	1	1.0	-	-	1	1.2	0	0.0	-	-

CH: Child Handbook; MoH: Ministry of Health; PHC: Primary Health Care; MAC: medium and high complexity services; UBS: Primary Health unit.

^a^ Nursing students.

^b^ University and digital/phone.

### Compilation and Analysis of Results

- Characterization of studies: study type – 1 (primary/secondary) and 2 (scientific articles/literature review articles/dissertations/theses) –; study design (as described by the authors); approach to CH; nomenclature given to the CH by the authors (adopted by the MoH/no and authors explicitly stated in the references that it was the MoH’s CH/no); publication year; region of Brazil (North/Northeast/Central-West/Southeast/South); population (family members or caregivers/healthcare professionals/both/others); scenario (Primary Health Care – PHC/medium and high complexity services – MAC/community/education/social assistance/more than one scenario); and location (primary health unit – UBS)/domicile/vaccination campaign/outpatient/school/hospital/more than one location/others).
- Results: the results of studies that used CH as a data source were briefly described, and those that used CH as an instrument for data collection, as an object of study, or that mentioned CH in the results were detailed (barriers and facilitators to CH use and limitations related to CH). The last two were presented together as they had similar results.

## RESULTS

**Figure 1:**
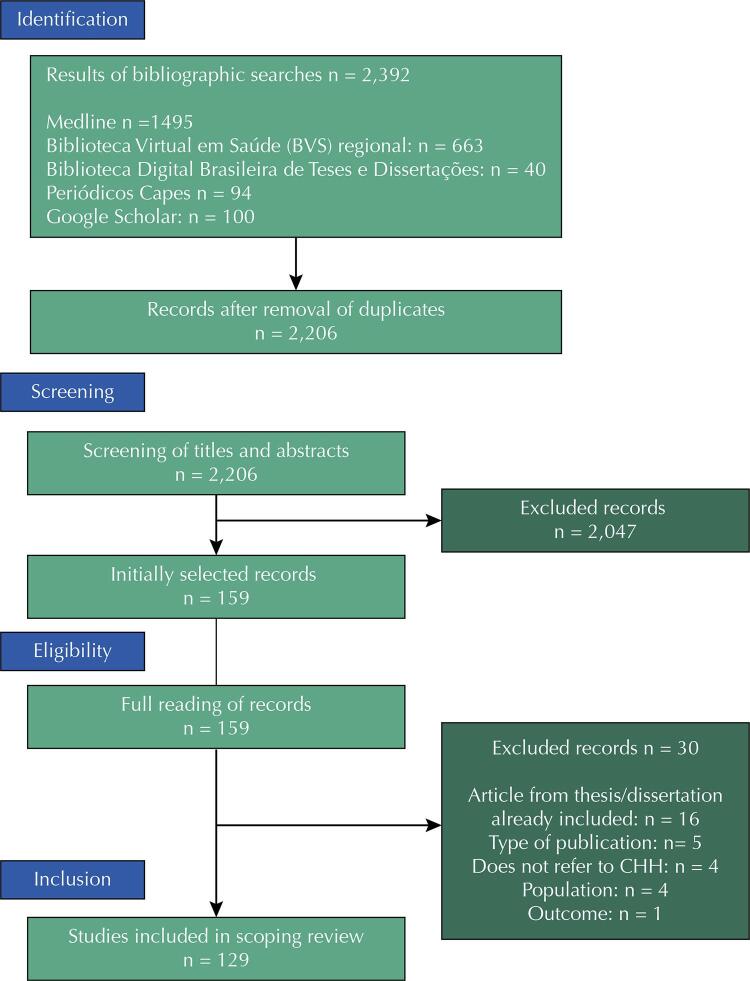
Scoping review flowchart with search and screening procedures - CPAPI, 2022.

A total of 2,206 records were retrieved after removing duplicates (Figure 1 – Prisma^15^flowchart). After screening, 159 studies were fully assessed, of which 129 were included and 30 were excluded (Supplementary Table 3^c^).

Capes: Coodenação de Aperfeiçoamento de Pessoal de Nível Superior; CHH: Child Health Handbook.

### Characterization of Studies

Ninety-two studies (71.3%) were articles published in scientific journals, including eight literature reviews; 30 were dissertations (23.3%) and seven were theses (5.4%) (Table 1). Most authors (60%, n = 75)^7,16^reported using a cross-sectional design. One hundred and thirteen studies (87.6%)^7,16^used primary data, 9.3% used secondary data^11,12,62,132^, and 3 .1% used both^34,43,68,141^.

Sixty studies (47.5%) used CH as a data source and 47 (36.4%) used it as a study object. In 11 (8.5%), CH was mentioned only in the results, and in four (3.1%), it was used as an instrument for data collection (Table 1). The remaining 7 refer to an article and six dissertations that used more than one approach to CH. Six of them use CH as a data source and also as an instrument for data collection (n = 3)^23,29,57^, object of study (n = 1)^16^or citation in the results (n = 2)^21,24^. In a dissertation^141^, the CH is cited in the results and is also an object of study (Table 1).

**Figure 2:**
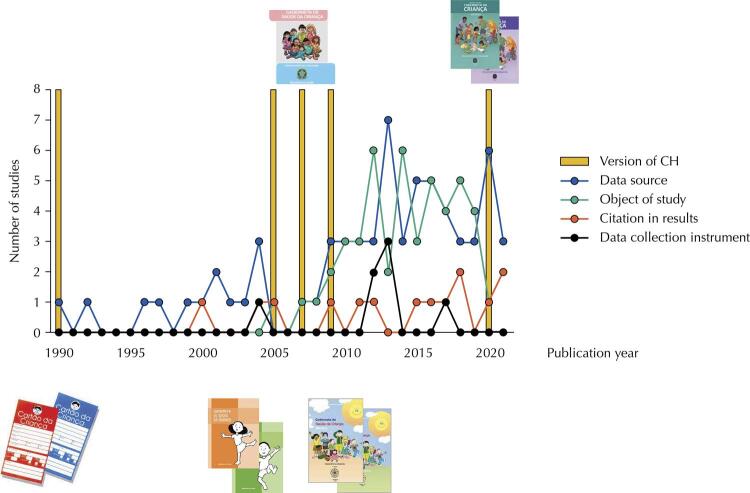
Temporal distribution of publications according to the approach to the Child Handbook (CH) and year of publication of its different versions - CPAPI, 2022.

Thirty-seven studies (28.7%)^20,26,30,31,37^used the terminologies “vaccine card”, “vaccination card”, “vaccination handbook”, “vaccine handbook”, and “vaccination record”, while those adopted by the MoH were “Child Card”, “CHH” or “CH” (Table 2). In 41.9% (n = 54) ^20,26,30^of the cases, the authors did explicitly state that the instrument used referred to the MoH’s CH (Table 2). The publication year ranged from 1990 to 2021, with a higher concentration from 2011 onwards (n = 98; 76%) (Figure 2).

In 75% (n = 81) ^7,17,22^of the studies, excluding review articles, family members or caregivers of children comprised the population from which data was collected (Table 2).

The Northeast was the region where 42.8% of the studies were conducted^7,16,17,21^, followed by the Southeast (30.8%)^19,20,24,26,31,33,34,36,37,40,41,43,50,54,55,60,61,63,64,68,79,84, 89, 94,95,97,103,105,107,111^. The main scenario of the studies is PHC (58.2%; n = 71)^7,11,12,16,17,20,22^. About one-third of the studies were carried out in UBS (32.5%; n= 38)^20,22,23,25,26,28,29,36,42,46,50,57,62,68,75^ (Table 2).

### Studies’ Approach to the CH

Table 1 describes the types of study according to their approach to CH.

### Data Source

The main data extracted from the CH were related to vaccination (62.3%, n = 38)^20,26,31,37^, followed by anthropometric data (31,2%, n = 19)^16,21,23,24,32,51,54,57,61,66,68,71,85,100,101, 103,105,109,118^, pre-partum, delivery, and birth period (19.7%, n = 12)^23,44,51,54,57,66,68,103,105,109,117,119^, iron and vitamin A supplementation (6.6%, n = 4)^30,45, 85,87^ and CD milestones (4.9%, n = 3) ^57,68,101^.

Most of the CH belonged to infants (< 2 years: 43.8%, n = 28) ^21,23,24,39,41^, infants and preschoolers (< 6 years old; 34.4%, n = 22)^16,20,26,29,30,38,44,45,47^or from infants to school-age children (< 10 years; 9.4%, n = 6)^40,50,58,71,115,121^. Some studies evaluated the CH of schoolchildren (≥ 6 and < 10 years; 6.3%, n = 4)^32,69,70,105^or broader age ranges (between 2 and 18 years, < 12 years or ≤ 18 years; 6, 3%, n = 4)^18,31,37,64^.

CH-related limitations^16,37,38,41^ were extracted from these studies: the fact that the caregiver did not have or was not carrying the CH on the day of the interview (prevalences ranging from < 5%^41^to 71%^48^) and incompleteness and illegibility^134^ of the data.

### Object of Study and Citation in Results

Of 102 investigated outcomes, the main ones were completion of the CH (n = 44, 43.1%)^7,11,12,16,17,21,22,24,25,27,33^ and factors associated with completion (n = 23, 22.5%)^7,17,22,33,35, 36,53,59,60,63,65,77,80,81,84,88,97,107,129,131,137,139,140^ (Table 2).

### Completion of the CH

The studies pointed out weaknesses in filling out all items of the CH^11,12,23,57,113,132,138,139^. Considering different criteria to define adequacy, the completion of the items varied between 18.9% and 70%^16,17,22,35,59,60,63,129,141^. Higher caregiver/mother education^17,59,63^, caregiver receiving guidance on CH^59,60,63^, caregiver/mother taking notes in the CH^35,60^, younger children^35,63^, main caregivers being parents or grandparents^59^, higher number of well-child appointments^35^, children followed by general practitioners^63^, children born at term^60^, attendance prenatal care^59^and prenatal care in the SUS^59^were factors associated with better completion of the CH (Box).

**Box:** Variables related to the use and/or completion of the Child Handbook – CPAPI, 2022.

Factors related to greater use and/or completion of the CH		
**Contextual features** CH availability^21,25,36,62,88,96,112,127,129,137,140^; urban region^21^; completion of weight^16^; trainings on the CH^11,16,21,22,25,35,53,88,90,96,99,112,127,129,130–132,137,139,140^; adequate work demand^90,91,99,113,127,129,137–140^; non-bureaucratic work process^60,90,99,139^; adequate number of professionals in the service^16,90,96,99,129^; availability of materials (scale, measuring tape, etc.)^11,16,99,129^; and lower diversity of handbook^22,130^.		
• Higher schooling of the mother/caregiver^17,59,63,96,132,138^ • Primary caregiver being parent or grandparent^59^ • Prenatal care attendance^59^ • Prenatal care at SUS^59^ • Mother/caregiver taking notes on CHH^35,60^	• Younger^35,63^ • Born at term^60^ • Higher number of well-child appointments^35^	• Work in PHC (vs. MAC)^33,90,113,129^ • Children followed by general practitioners (vs pediatricians)^63^ • Communication between healthcare professionals^90,107,122,127,137,139,140^ • Knowledge about CH ^11^,^21^,^35^,^53,^ ^77–79,88,90,95,99,113,127,128,130–132,137,139,141^ • Provide guidance and dialogue with caregivers about the CH^53,59,60,63,91,112,132,138,139^
**Factors related to completion of anthropometric measurements in the CH**		
• Higher schooling of the mother/caregiver (weight^84,88^) • Higher income (weight^83,88^; height chart^83^) • Lower income (weight^7^) • Older (≥ 20 years - weight^84,88^) • Younger (< 35 years - weight^53^; height^53^) • First-time mother (weight^88^) • Mothers who do not work outside the home (weight^7^)	• Older (weight^53,88^; weight chart^65^; height^53^) • Younger (weight^83^; height chart^83^) • Higher number of well-child appointments (weight^97^) • Well-child visits in FHS (vs traditional, height^53^)	Provide guidance and dialogue with caregivers about the CH^84,131^
**Factors related to completion of CD milestones in the CH**		
• Higher schooling of the mother/caregiver^7^ • Higher income^24^ • First-time mother^7,65,88^ • Not residing in the area covered by the CHW^7^	• Female^88^ • Older^7^ • Non-anemic^24^	Provide guidance and dialogue with caregivers about the CH^131^
**Factors related to the mother’s reading of the CH and bringing it to appointments**		
READING • Higher schooling of the mother/caregiver^53,34^ • White ethnicity^34^ • Mother not being the head of household^34^ • Having ≥ 6 prenatal care visits^34^	READING • Older^53^ BRINGING • Younger^94^	BRINGING • Requesting the CH during appointments^84,96^ • Taking notes in the CH^84,96^ • Provide guidance and dialogue with caregivers about the CH^9^
**Family members and caregivers**	**Children**	**Healthcare professionals**

FHS: Family Health Strategy; CH: Child Handbook; PHC: Primary Health Care; MAC: medium and high complexity services; CD: child development; CHW: community health worker.

The monitoring items with the highest prevalence of completion were vaccination and anthropometric measures. Completion of vaccination items varied between 91.8% and 100%^17,59,60,63,80,82,84,94^. Filling in the child’s weight item ranged from 2% to 96.3%^16,17,21, 25,27,34,53,59,83,86,88,94,97,125^and these measures were transposed to the chart between 9% and 100% of the time^21,22,34,63,65,84,88,92,94,97,137^. Lower prevalences were found for height completion, ranging from 19% to 66.2%^16,53,59,94,137^, with the height chart completed from 8.9% to 100% of cases^22^. Even lower completion prevalences were found for head circumference, ranging from 9.7% to 43.1%^88,137^, and the chart of this measure (21.9% to 72.7%)^22,59,92^.

Completion of developmental milestones varied between 0% and 72.7%^7,16,17,24,27,34,35,59,60,65,82,84,88, 92, 94,98,122,136,137^. Poorer prevalences or absence of records were attributed to iron and vitamin A supplementation, as well as auditory, ocular, and oral health^22,33,60,63,82,84,141^.

### Factors Associated with CH Use

The factors associated with inadequate use of CH include the poor knowledge of healthcare professionals about CH^11,21,35,53,77^, and many studies highlight the lack of training on the CH directed towards these professionals ^11,12,16,21,22,25,35,53,88,90,96,99,112,127,129^.

CH is more commonly used in outpatient care, not being highly valued in the hospital setting ^33,90,113,129^. Although healthcare professionals recognize that the lack of CH records and discontinuation of CH use can hinder the monitoring of the child’s health^127,130,139^, they mainly understand CH as an instrument for recording vaccination and growth^80,90, 96,107,127,129^. In the statements of these professionals, CH also appears as something that allows and guides the child monitoring by different services^90,99,129^and stimulates communication with family members^78,90,96,98,127^; however, these uses are not observed in practice^18,60, 90,93,96,107,127,129,132,140^. Community health workers (CHW) are seen as a strategic point for working with CH^7^, but they do not feel valued by the population, as they observe resistance from mothers when requesting the CH for consultation^94^. A study that aimed to describe the practices of home visits by CHW according to region and location of UBS identified that the Northeast region had the highest percentages of verification of the “vaccination card” (51%)^126^.

Work process-related factors, such as lack of CH^21,53,88,96,112,127,129,137,140^, high demand for care^90,91,99,113,127,129,137^, and others, also influence CH completion (Box). Among the states of the Northeast macro-region, 46.9% of children aged 0 to 2 years received the CH, ranging from 36.8% (Sergipe) to 58.8% (Ceará)^62^. Only 50–55% of children receive the CH in the maternity ward^21,25^.

Healthcare professionals claim that there is a lack of interest and little engagement of family members and caregivers in CH use – as they do not read or bring the CH to the appointments^90,91,94,96,112,129,132,137,139,140^–, but they believe that CH enables mothers to understand their child’s health^127^. They also argue that family members should require the completion of CH^90^, co-responsibilizing themselves for its use^90,99^. On the other hand, they recognize that caregivers’ non-use of CH may be related to not receiving guidance for it^91,112,132,139^. The percentage of families or caregivers who received guidance on CH ranged from 33% to 64.3%^33,35,60,63,84,94,129^.

On the other hand, despite recognizing the CH as a child’s document and a technology to assist in caring for their children^33,96,104^, family members and caregivers feel excluded from the process, as there is no dialogue about the instrument nor encouragement to read and use it^96,140^. Nevertheless, between 80.3% and 88% of the mothers reported having read the CH^34,35,53^. It is noteworthy that between 18% and 26% of mothers make notes in their children’s handbooks^35,60,63,84^, which is associated with better completion rates^35,60^.

The percentage of family members and caregivers who had and carried the CH at the time of the study varied between 46.9% and 100%^7,21,22,24,25,27,36,53,59,83,84,86,94 ,97, 125^. The percentage of caregivers who reported bringing the CH to appointments ranged from 76% to 93.5%^35,84,94^. However, some studies have identified that 52% of mothers were instructed to bring the CH to appointments^94^, from 70% to 86.5% of healthcare professionals requested CH during the appointment^21,25,35^, 49% of the professionals took notes on CH during the appointment^94^, and 25% of family members and caregivers perceived indifference from the doctor regarding the CH^84^. According to family members and caregivers, not receiving guidance from healthcare professionals regarding CH^53,138^and the difficulty in understanding the information contained therein^96,132,138^are factors associated with reduced use of the CH by these actors.

Box represents the factors related to the use and/or completion of the CH.

### Intervention Studies

In the four intervention studies identified^93,94,112,122^, the strategies to expand the use of the CH include collective monitoring using the CH^93^, reminders added to the child’s medical record for the professional to complete the CH^122^, and the creation of spaces for dialogue about the CH^112^. One of the studies^94^ warns that training may not have the expected impact, and it is necessary to plan strategies to reach the target audience and involve managers. Lack of adequate space, support and organization, work overload and lack of participation of healthcare professionals were barriers to these actions^93,94,112,122^.

### Data Collection Instrument

One study used the weight-for-age curve to evaluate the nutritional status of children aged 6 to 60 months using two methodologies (Waterlow criteria = 42.9% and CH chart = 35.6%), concluding that the prevalence of children with nutritional problems was similar and high^29^.

Six studies used the CH’s surveillance instrument of CD^19,23,28,46,57,89^. One study evaluated the neuropsychomotor development of children aged 0 to 18 months^28^ using the CH milestones, showing that 53% of them presented all the expected milestones for their age. Another study^46^ evaluated the accuracy of the CH instrument as compared to the Bayley III screening test. The CH surveillance instrument presented moderate sensitivity (57.1%), specificity (69.4%), accuracy (64%), positive predictive value (59.5%), and negative predictive value (67.3%), failing to identify 43% of children at risk or with probable developmental delay. Two studies^19,89^ found low agreement between the CH surveillance instruments and the Integrated Management of Prevalent Childhood Illness (IMPCI) instruments for children aged between two months and two years: agreement of 0.34 (Kappa coefficient = -0.12; p = 0.98)^19^and 31.6% in CH and 34.1% in IMPCI with delayed CD (Kappa coefficient = 0.27)^89^. Another found low agreement between the Alberta Infant Motor Scale (AIMS) and CH (Kappa coefficient = 0.077 to 0.096; p = 0.000)^23^. No agreement was found between the CH instrument and the Harris Infant Neuromotor Test (HINT) scale (Kappa coefficient = -0.01)^57^.

Studies that seek to compare the CH CD surveillance instrument with other instruments encounter some difficulties: (i) the number of milestones evaluated^46^and the indicators of CD alterations are based on different criteria^57,89^; (ii) the scoring and classification methods are different, which may impact the results^46^; (iii) the CH instrument does not mention the need to correct chronological age for preterm children^46^; (iv) there is no definition of the time that the evaluator should wait or number of attempts for performing a certain milestone^46^; (v) the CH instrument advances CD milestones that should be required at later age groups, leaving doubts regarding the child’s classification^46,89^; and (vi) vague explanation and imprecise language in the design of the CH instrument^46,128^.

## DISCUSSION

As far as we know, this is the first scoping review that compiles all scientific production published between 1990 and July 2021 on the CH in Brazil. A significant number of studies were identified (n = 129), and almost half of them used the CH as a data source, mainly for vaccination data. The other half took the CH as an object of study, citing it in the results or using it as a data collection instrument. In this case, the main outcomes investigated were the completion and factors associated with completing the CH, in which weaknesses were identified in completing all items, except for vaccination. Contextual characteristics related to children, family members and caregivers, as well as professionals, were related to the use and/or completion of the CH.

The CH has been contributing to the study of vaccination coverage^20,26,31,37^and, to a lesser extent, for the study of children’s nutritional status^16,21,23,24,32,51,54,57,61,66,68,71,85,100,101,103,105,109,118^. That is, as the CH is properly filled out, the possibilities of its use in analysing different health outcomes increase. The use of an inappropriate nomenclature demonstrates that the authors may be limiting the CH to its initial use^11,80,141^ of recording vaccination, and this view seems to be perpetuated and transmitted to caregivers, not favoring the change to an expanded view of the CH. The CH is also an instrument intended for empowering families to care for their children, but these actors have not been sufficiently involved in the care process. Despite these barriers, Brazilian studies indicate a positive association between the use of the CH and better outcomes, such as the child’s nutritional status^86^and CD^142^.

Almost half of the studies were conducted in the Northeast region. A national study on home visits identified higher percentages of verification of the “vaccination cards” (51%) and weighing of children (41.3%) in this region^126^, which may indicate that child health monitoring activities are a priority in the scope of the Northeastern PHC. PHC was the scenario for most of the studies, in line with its role as the coordinator of care in health care networks^143^. Consequently, the UBS were the preferred locations for conducting studies, as a viable and cost-effective alternative for research on child health. It is worth highlighting the use of vaccination campaigns as an alternative for carrying out surveys on health, nutrition, and CD in Brazil^146,148^. However, little emphasis was given to the role of other points of care^139^, such as maternity wards, which are crucial in the distribution and initial completion of the CH.

The interest in studying the use of the CH seems to have started in 2005, coinciding with the launch of the CHH, when the document began to be used as an instrument for surveillance and comprehensive monitoring of child health more emphatically. The main outcome investigated by studies that used the CH as an object of study or cited it in their findings was its completion and factors associated with completion. It is, therefore, the obtaining of a diagnosis on the use of this tool, which can help in defining strategies to improve the currently restricted scenario of its use. However, the need for the inclusion of the CH theme in processes of continuing education for the incorporation of this instrument as a guide for health practices is clear^11,21,35,53,77^. Also, the production in the field of implementation research is small, and few studies advance in proposing intervention models to solve the problem^93,94,112,122^.

The incorporation of CD surveillance into the work process of PHC using the CH is yet another challenge^122,136^. The proposal to carry out a physical examination of the child and identify CD risk factors (biological, social, and environmental), as well as observing developmental milestones, make the CH a tool to support professionals in CD surveillance^144^. Therefore, studies that aimed to validate the CH’s CD milestones as a screening tool should be interpreted with caution since the purpose of the MoH is to use these milestones along with other information to identify children with possible delays. Thus, more studies are needed considering all the aspects raised in the CH for CD surveillance and its purpose of global CD assessment.

The scope of the bibliographic search conducted and the inclusion of grey literature are strengths of this review. On the other hand, the exclusion of conference abstracts and undergraduate course papers, as well as limited consultation to the first ten pages of Google Scholar and the non-availability of CH reports from studies funded by the MoH may be considered limitations.

The CH, in its multifunctionality, can be a formative tool and support for professionals working in child health and guidance to caregivers, and the data generated with its completion can contribute to the development of public policies involving early childhood. Nevertheless, the findings of this review reinforce the need to better understand which factors affect the distribution of the CH to the population and how to improve this process. They also point to the need to understand its use and completion by the different points of care and sectors, in the different Brazilian contexts. The need for studies that evaluate training models for healthcare professionals to use the CH in the context of continuing health education and interventions that encourage caregivers to use the CH is evident. Thus, researchers, policymakers, managers, healthcare professionals, and other sectors should seek in the CH a way to effectively systematize and monitor child health care, strengthening this important strategy inserted in policies to promote healthy growth and development and reduce child morbidity and mortality.

## Footnotes

a Available from: https://drive.google.com/file/d/17ij9gme-VrY-ohObwV6rF1mWqmESGnVA/view?usp=sharing

b Available from: https://drive.google.com/file/d/1iIuSqlqN41uKr1pQack3pcPDY83Lv9eW/view?usp=sharing

c Available from: https://drive. google.com/file/d/1krai83iYTd5o HGItjVArx3Vzdn_hweRl/view? usp=sharing
