# Supplementary material for: Studies on the Child Handbook in Brazil: a scoping review
Source: Rev Saude Publica. 2023 Jul 31;57:48. doi: 10.11606/s1518-8787.2023057004733 (PMC10392774; doi:10.11606/s1518-8787.2023057004733)
Supplement: Translation

# Translation: Estudos sobre a Caderneta da Criança no Brasil: uma revisão de escopo

**Authors:** Juliana Araujo Teixeira, Cintia de Freitas Oliveira, Maritsa Carla de Bortoli, Sonia Isoyama Venâncio

**Affiliations:** I Brazil Insper. Centro Brasileiro de Pesquisa Aplicada à Primeira Infância. São Paulo, SP, Brazil; II Brazil Secretaria de Estado da Saúde. Instituto de Saúde. São Paulo, SP, Brazil

**Keywords:** Saúde da Criança, Crescimento e Desenvolvimento, Avaliação de Processos em Cuidados de Saúde, Revisão

## Abstract

**OBJETIVO:**

Identificar e mapear de forma sistemática os estudos que envolvem a Caderneta da Criança (CC) no Brasil.

**MÉTODOS:**

Revisão de escopo utilizando a metodologia proposta pelo Instituto Joanna Briggs. Foram consultadas as bases de dados PubMed, BVS Regional, Biblioteca Digital Brasileira de Teses e Dissertações, Periódicos Capes e Google Scholar. Incluíram-se estudos primários e secundários que mencionavam o uso da CC, sem restrições quanto ao delineamento, ano de publicação ou população, publicados em português, inglês ou espanhol e literatura cinzenta (teses e dissertações).

**RESULTADOS:**

Foram incluídos 129 estudos, em sua maioria descritivos, publicados como artigos científicos. A região Nordeste, a população de cuidadores das crianças e o cenário da atenção primária foram observados na maioria dos estudos; 47% dos estudos utilizou a CC como fonte de dados, a maioria sobre vacinação. Apesar dos diferentes critérios para definir adequação de preenchimento da CC, os estudos identificaram fragilidades no preenchimento de todos os seus itens, com exceção da vacinação. Características contextuais (como a disponibilidade da CC), das crianças (sexo, idade, prematuridade e consultas de puericultura), dos familiares e cuidadores (idade, escolaridade, renda, paridade, trabalho, pré-natal, ler, fazer anotações e levar a CC nas consultas) e dos profissionais (local de atuação, especialidade médica, comunicação no trabalho, conhecimentos sobre a CC, solicitar, orientar sobre e realizar anotações na CC) foram relacionadas ao uso e/ou preenchimento da CC.

**CONCLUSÕES:**

Os resultados reforçam a necessidade de melhor compreender quais fatores afetam a distribuição da CC à população, assim como apontam para a necessidade de compreender o seu uso e preenchimento pelos diversos pontos de atenção. Fica clara a necessidade de estudos de intervenção para que a implementação desse instrumento seja aprimorada e de incluir capacitações sobre a caderneta no contexto da educação permanente em saúde.

## INTRODUÇÃO

O progresso mundial na redução da mortalidade infantil é notável^1^. Neste momento, esforços vêm sendo direcionados não apenas para reduzir a mortalidade, mas também para promover o desenvolvimento pleno de todas as crianças^2^. Dessa forma, ações educativas junto às famílias, o acompanhamento e registro de dados sobre a saúde da criança e a integração das ações entre os serviços são fundamentais^3^.

Instrumentos para registros de informações, como as cadernetas de saúde, têm sido utilizados em mais de 163 países com o objetivo de melhorar a saúde materna e infantil^4,5^. Uma revisão sistemática identificou que as cadernetas de saúde de gestantes e crianças são eficazes para a melhora da saúde^6^, com impactos positivos na promoção da busca de cuidado, melhor conhecimento e práticas de cuidado, cuidado domiciliar encorajado para doenças infantis, mortalidade e morbidade infantil reduzidas e facilitação do cuidado contínuo.

No Brasil, o Cartão da Criança foi criado em 1984 pelo Ministério da Saúde (MS), em substituição à Caderneta de Vacinações, para incluir o acompanhamento do crescimento e do desenvolvimento infantil (DI) de crianças de 0 a 5 anos^7,8^. Esse instrumento passou por diversas atualizações e, em 2005, foi criada a Caderneta de Saúde da Criança (CSC), como um instrumento de acompanhamento integral da saúde da criança. Em 2021, foi lançada a Caderneta da Criança (CC), destinada a todas as crianças nascidas em território brasileiro, que prevê o acompanhamento de crianças de 0 a 10 anos. Propõe-se que essa nova versão seja utilizada pelas famílias e por profissionais de saúde e de outros serviços que atendem a criança, como os da educação e assistência social, facilitando a integração das ações^9,10^. A primeira parte da CC é direcionada às famílias e a segunda, aos registros dos profissionais para o acompanhamento da criança, contando com a inclusão de um instrumento para a triagem do autismo^10^.

A CC é o principal instrumento para a vigilância do crescimento e desenvolvimento saudáveis segundo a Política Nacional de Atenção Integral à Saúde da Criança (PNAISC)^3^. Apesar do seu potencial, e de ser uma ação instituída pelo MS, de abrangência nacional, estudos destacam uso e preenchimento inadequados da CC, sem consenso quanto aos fatores associados a esse uso ou intervenções que possam melhorá-lo^11,12^. Diante disso, essa revisão visa mapear os estudos sobre a CC do MS que tenham analisado seu uso, barreiras e facilitadores e intervenções voltadas à sua utilização, a fim de oferecer informações para a sua efetiva implementação.

## MÉTODOS

### Delineamento do Estudo

Realizou-se uma revisão de escopo, método sistemático para o mapeamento da produção científica sobre determinado tema com o objetivo de identificar conceitos e lacunas de pesquisa^13^, utilizando a metodologia do Instituto Joanna Briggs^13^. A seguinte pergunta foi considerada, a partir do acrônimo PCC (população, conceito e contexto): Qual é a produção científica sobre a CC utilizada por profissionais da saúde, gestores, familiares e cuidadores no Brasil? O protocolo de pesquisa foi publicado no repositório Zenodo (https://sandbox.zenodo.org/record/891923#.YQhK445KhPY).

### Critérios de Elegibilidade

Incluíram-se estudos primários e secundários que mencionavam o uso da CC no contexto brasileiro, sem restrições quanto ao delineamento do estudo, ano de publicação ou população, publicados em português, inglês ou espanhol. Da literatura cinzenta foram incluídas teses e dissertações e excluídos resumos de congresso e trabalhos de conclusão de curso. Os trabalhos que não explicitavam que o instrumento utilizado se referia à CC só foram incluídos se a descrição do estudo não deixasse dúvidas de que se tratava do instrumento do MS.

### Busca e Seleção dos Estudos

Após mapeamento de termos relacionados à pergunta de pesquisa e discussões entre especialistas, uma estratégia de busca foi construída para o PubMed e posteriormente adaptada por duas bibliotecárias para as demais plataformas de pesquisa. A Tabela suplementar 1^a^ mostra as datas, os termos, bases e as estratégias utilizadas.

A triagem dos títulos e resumos e a leitura dos textos completos foram feitas por duas revisoras independentes utilizando a plataforma Rayyan^14^, e as divergências resolvidas por uma terceira. Em ambas as etapas, uma amostra de estudos foi selecionada no início do processo para a calibração dos critérios de inclusão e exclusão.

### Extração dos Dados

Elaborou-se uma planilha de extração (Tabela suplementar 2^b^) e três artigos foram selecionados para calibração. Os estudos foram extraídos de forma independente por duas revisoras e as divergências resolvidas por uma terceira. Foram coletados: (i) caracterização dos estudos, (ii) resultados, (iii) barreiras e facilitadores à utilização da CC e (iv) limitações relacionadas à CC. Para cada estudo incluído foi identificada a abordagem sobre a CC: (i) fonte de dados (estudos que coletavam informações de interesse dos autores sobre a criança, por meio da CC, por exemplo dados de vacinação); (ii) objeto de estudo (objetivo era estudar a CC, por exemplo, seu preenchimento e fatores associados); (iii) citação em resultados (citavam a CC somente nos resultados); e (iv) instrumento para coleta de dados (utilizavam algum instrumento da CC para a coleta de dados, por exemplo o instrumento de vigilância do DI).

### Compilação e Análise dos Resultados

(i) Caracterização dos estudos: tipo de estudo – 1 (primário/secundário) e 2 (artigos científicos/artigos de revisão de literatura/dissertações/teses) –; delineamento de estudo (conforme descrito pelos autores); abordagem sobre a CC; nomenclatura dada à CC pelos autores (adotada pelo MS/não e autores explicitavam nas referências ser a CC do MS/não); ano de publicação; região do Brasil (Norte/Nordeste/Centro-Oeste/Sudeste/Sul); população (familiares ou cuidadores/profissionais de saúde/ambos/outros); cenário (atenção primária à saúde – APS/serviços de média e alta complexidade – MAC/comunidade/educação/assistência social/mais de um cenário); e local (unidade básica de saúde – UBS)/domicílio/campanha de vacinação/ambulatório/escola/hospital/mais de um local/outros).

(ii) Resultados: foram descritos sucintamente os resultados dos estudos que utilizaram a CC como fonte de dados e detalhadamente (barreiras e facilitadores à utilização da CC e limitações relacionadas à CC) aqueles que utilizaram a CC como instrumento para coleta de dados, como objeto de estudo ou que citaram a CC nos resultados, sendo que os dois últimos foram apresentados conjuntamente por apresentarem resultados semelhantes.

## RESULTADOS

Foram recuperados 2.206 registros após a remoção das duplicidades (Figura 1 – fluxograma Prisma^15^). Após a triagem, 159 estudos foram avaliados na íntegra, dos quais 129 foram incluídos e 30 excluídos (Tabela suplementar 3^c^).

**Figura 1:** Fluxograma da revisão de escopo com os procedimentos de pesquisa e triagem – CPAPI, 2022.

Capes: Coordenação de Aperfeiçoamento de Pessoal de Nível Superior; CSC: Caderneta de Saúde da Criança.

### Caracterização dos Estudos

Noventa e dois estudos (71,3%) são artigos publicados em revistas científicas, oito deles são revisões da literatura; 30 dissertações (23,3%) e sete teses (5,4%) (Tabela 1). A maioria dos autores (60%, n = 75)^7,16^ reportaram ter utilizado um delineamento transversal. Cento e treze estudos (87,6%)^7,16^ utilizaram dados primários, 9,3% dados secundários^11,12,62,132^ e 3,1% ambos^34,43,68,141^.

**Tabela 1:** Classificação dos estudos quanto à abordagem sobre a Caderneta da Criança e tipo de estudo – CPAPI, 2022.

Abordagem sobre a CC	Tipo de estudo	n	%	Citações
Fonte de dados	Artigo	50	75,8	18, 20, 26, 30, 31, 37, 39–43, 45, 47–49, 52, 55, 56, 58, 61, 64, 68–76, 85, 87, 100, 102, 105, 106, 109, 110, 114, 115, 118, 119, 121, 123, 124, 134, 142–145
	Artigo de revisão	0	0	-
	Dissertação de mestrado	12	18,2	16, 21, 23, 24, 44, 50, 54, 57, 66, 67, 108, 111
	Tese de doutorado	4	6,1	32, 51, 101, 103
Objeto de estudo	Artigo	24	49	7, 17, 27, 33, 36, 53, 59, 63, 65, 77, 79–84, 86, 91, 92, 95, 112, 116, 130, 131
	Artigo de revisão	6	12,2	132, 136–140
	Dissertação de mestrado	16	32,7	16, 22, 35, 60, 88, 90, 93, 96, 107, 122, 125, 127, 129, 133, 135, 141
	Tese de doutorado	3	6,1	34, 94, 99
Citação em resultados	Artigo	7	50	62, 78, 97, 98, 104, 126, 128
	Artigo de revisão	2	14,3	11, 12
	Dissertação de mestrado	5	35,7	21, 24, 25, 113, 141
	Tese de doutorado	0	0	-
Instrumento de pesquisa	Artigo	4	57,1	19, 28, 29, 89
	Artigo de revisão	0	0	-
	Dissertação de mestrado	3	42,9	23, 46, 57
	Tese de doutorado	0	0	-

Total de abordagens sobre a Caderneta da Criança: n = 136; artigo: n = 84 (65,1%); artigo de revisão: n = 8 (6,2%); dissertação de mestrado: n = 30 (23,3%) e tese de doutorado: n = 7 (5,4%).

Sessenta estudos (47,5%) utilizaram a CC como fonte de dados e 47 (36,4%) como objeto de estudo. Em 11 (8,5%), a CC foi citada somente nos resultados e em 4 (3,1%) utilizada como instrumento para a coleta de dados (Tabela 1). Os sete restantes referem-se a um artigo e seis dissertações que utilizaram mais de uma abordagem quanto a CC. Seis deles usam a CC como fonte de dados e também como instrumento para coleta de dados (n = 3)^23,29,57^, objeto de estudo (n = 1)^16^ ou citação nos resultados (n = 2)^21,24^. Em uma dissertação^141^, a CC é citada nos resultados e também é objeto de estudo (Tabela 1).

Trinta e sete estudos (28,7%)^20,26,30,31,37^ utilizaram as terminologias “cartão de vacinas”, “cartão de vacinação”, “carteira de vacinação”, “carteiras vacinais” e “caderneta de vacinação”, enquanto as adotadas pelo MS eram Cartão da Criança, CSC ou CC (Tabela 2). Em 41,9% (n = 54)^20,26,30^ dos casos, os autores não explicitaram que o instrumento utilizado se referia à CC do MS (Tabela 2). O ano das publicações variou entre 1990 e 2021, com maior concentração a partir de 2011 (n = 98; 76%) (Figura 2).

**Tabela 2:** Características dos estudos segundo abordagem sobre a Caderneta da Criança – CPAPI, 2022.

Variável	Total		Abordagem dos estudos sobre a CC							

			Fonte de dados		Objeto de estudo		Citação em resultados		Instrumento de pesquisa	
				
	n	%	n	%	n	%	n	%	n	%
									
	136	100,0	66	47,0	49	39,0	14	10,3	7	3,7
Nomenclatura adotada pelo MS										
Não	37	28,7	33	50,0	2	4,1	2	14,3	0	0,0
Sim	92	71,8	33	50,0	47	95,9	12	85,7	7	100,0
CC/MS explicitada pelos autores										
Não explicitada	54	41,9	47	71,2	4	8,2	3	21,4	0	0,0
Explicitada	75	58,1	19	28,8	45	91,8	11	78,6	7	100,0
Região do Brasil										
Norte	7	5,3	3	4,5	3	6,1	1	6,7	0	0,0
Nordeste	57	42,8	30	44,8	20	40,8	7	46,7	6	66,7
Centro-Oeste	17	12,8	7	10,4	7	14,3	2	13,3	1	11,1
Sudeste	41	30,8	21	31,3	15	30,6	4	26,7	2	22,2
Sul	11	8,3	6	9,0	4	8,2	1	6,7	0	0,0
População										
Familiares e cuidadores	81	75,0	54	93,1	21	53,8	4	33,3	5	100,0
Profissionais de saúde	17	15,7	1	1,7	12	30,8	6	50,0	0	0,0
Ambos	9	8,3	3	5,2	5	12,8	2	16,7	0	0,0
Outros^a^	1	0,9	0	0,0	1	2,6	0	0,0	0	0,0
Cenário										
APS	71	58,2	24	37,5	34	77,3	13	92,9	6	85,7
MAC	16	13,1	13	20,3	3	6,8	0	0,0	0	0,0
Comunidade	16	13,1	15	23,4	1	2,3	0	0,0	0	0,0
Educação	10	8,2	8	12,5	2	4,5	0	0,0	0	0,0
Assistência social	1	0,8	1	1,6	0	0,0	0	0,0	0	0,0
Mais de um cenário	8	6,6	3	4,7	4	9,1	1	7,1	1	14,3
Local										
UBS	38	32,5	14	22,2	14	33,3	8	66,7	6	85,7
Domicílio	26	22,3	22	34,9	3	7,1	2	16,7	0	0,0
Campanha de vacinação	8	6,8	2	3,2	5	11,9	1	8,3	0	0,0
Ambulatório	8	6,8	8	12,7	0	0,0	0	0,0	0	0,0
Escola	6	5,1	5	7,9	1	2,4	0	0,0	0	0,0
Hospital	5	4,3	4	6,3	1	2,4	0	0,0	0	0,0
Mais de um local	22	18,8	7	11,1	15	35,7	1	8,3	1	14,3
Outros^b^	4	3,4	1	1,6	3	7,1	0	0,0	0	0,0
Desfechos investigados										
Preenchimento da CC	44	43,1	-	-	37	44,0	9	45,0	-	-
Fatores associados ao @@preenchimento da CC	23	22,5	-	-	22	26,2	1	5,0	-	-
Percepções de profissionais de saúde sobre a CC	14	13,7	-	-	12	14,3	2	10,0	-	-
Aspectos relacionados à implementação	7	6,9	-	-	2	2,4	5	25,0	-	-
Conhecimentos de profissionais de saúde sobre a CC	5	4,9	-	-	4	4,8	1	5,0	-	-
Fatores associados a leitura da CC pela mãe	2	2,0	-	-	2	2,4	0	0,0	-	-
Inspeção da CC (vacinação) pelos profissionais de saúde	2	2,0	-	-	1	1,2	1	5,0	-	-
Fatores associados ao uso da CC	1	1,0	-	-	1	1,2	0	0,0	-	-
Fatores associados a levar a CC nas consultas	1	1,0	-	-	1	1,2	0	0,0	-	-
Ensino sobre a CC	1	1,0	-	-	1	1,2	0	0,0	-	-
Acesso à CC	1	1,0	-	-	0	0,0	1	5,0	-	-
Conhecimentos dos pais sobre a CC	1	1,0	-	-	1	1,2	0	0,0	-	-

CC: Caderneta da Criança; MS: Ministério da Saúde; APS: atenção primária à saúde; MAC: serviços de média e alta complexidade; UBS: unidade básica de saúde.

^a^Estudantes de enfermagem.

^b^Universidade e digital/telefone.

**Figura 2:** Distribuição temporal das publicações de acordo com a abordagem sobre a Caderneta da Criança (CC) e ano de publicação de suas diferentes versões – CPAPI, 2022.

Em 75% (n = 81)^7,17,22^ dos trabalhos, excluindo-se os estudos de revisão, familiares ou cuidadores de crianças compuseram a população a partir da qual os dados foram coletados (Tabela 2).

A região de realização de 42,8%^7,16,17,21^ dos estudos é a Nordeste, seguida da Sudeste (30,8%)^19,20,24,26,31,33,34,36,37,40,41,43,50,54,55,60,61,63,64,68,79,84,89,94,95,97,103,105,107,111^. O principal cenário dos estudos é a APS (58,2%; n = 71)^7,11,12,16,17,20,22^. Cerca de 1/3 dos estudos foram realizados em UBS (32,5%; n = 38)^20,22,23,25,26,28,29,36,42,46,50,57,62,68,75^ (Tabela 2).

### Abordagem dos Estudos sobre a CC

A Tabela 1 descreve os tipos de estudo de acordo com a abordagem em relação a CC.

### Fonte de Dados

Os principais dados extraídos da CC foram relativos à vacinação (62,3%, n = 38)^20,26,31,37^, seguidos de dados antropométricos (31,2%, n = 19)^16,21,23,24,32,51,54,57,61,66,68,71,85,100,101,103,105,109,118^, do período pré-parto, parto e nascimento (19,7%, n = 12)^23,44,51,54,57,66,68,103,105,109,117,119^, suplementação de ferro e vitamina A (6,6%, n = 4)^30,45,85,87^ e marcos do DI (4,9%, n = 3)^57,68,101^.

As CC pertenciam, em sua maioria, a lactentes (< 2 anos: 43,8%, n = 28)^21,23,24,39,41^, lactentes e pré-escolares (< 6 anos; 34,4%, n = 22)^16,20,26,29,30,38,44,45,47^ ou de lactentes à escolares (< 10 anos; 9,4%, n = 6)^40,50,58,71,115,121^. Alguns trabalhos avaliaram a CC de escolares (≥ 6 e < 10 anos; 6,3%, n = 4)^32,69,70,105^ ou faixas etárias mais abrangentes (entre 2 e 18 anos, < 12 anos ou ≤ 18 anos; 6,3%, n = 4)^18,31,37,64^.

Foram extraídas desses estudos as limitações relacionadas à CC^16,37,38,41^: o fato de o cuidador não ter ou não estar portando a CC no dia da entrevista (prevalências variando entre < 5%^41^ e 71%^48^) e a incompletude e ilegibilidade^134^ dos dados.

### Objeto de Estudo e Citação em Resultados

De 102 desfechos investigados, os principais foram o preenchimento da CC (n = 44, 43,1%)^7,11,12,16,17,21,22,24,25,27,33^ e fatores associados ao preenchimento (n = 23, 22,5%)^7,17,22,33,35,36,53,59,60,63,65,77,80,81,84,88,97,107,129,131,137,139,140^ (Tabela 2).

### Preenchimento da CC

Os estudos apontaram para fragilidades em relação ao preenchimento de todos os itens da CC^11,12,23,57,113,132,138,139^. Considerando diferentes critérios para definir adequação, o preenchimento dos itens variou entre 18,9% e 70%^16,17,22,35,59,60,63,129,141^. Maior escolaridade do cuidador/mãe^17,59,63^, cuidador receber orientação sobre a CC^59,60,63^, cuidador/mãe realizar anotações na CC^35,60^, crianças mais novas^35,63^, cuidadores principais serem pais ou avós^59^, maior número de consultas de puericultura^35^, crianças acompanhadas por médicos generalistas^63^, crianças nascidas à termo^60^, realização de pré-natal^59^ e pré-natal no SUS^59^ foram fatores associados ao melhor preenchimento da CC (Quadro).

**Quadro:** Variáveis relacionadas ao uso e/ou preenchimento da Caderneta da Criança – CPAPI, 2022.

Fatores relacionados ao maior uso e/ou preenchimento da CC		
**Características contextuais** Disponibilidade da CC^21,25,36,62,88,96,112,129,137,140^; região urbana^21^; preenchimento do peso^16^; capacitações sobre a CC^11,16,21,22,25,35,53,88,90,96,99,112,127,129,130,132,137,139,140^; demanda adequada de trabalho^90,91,99,113,127,129,137–140^; processo de trabalho não burocrático^60,90,99,139^; número adequado de profissionais no serviço^16,90,96,99,129^; disponibilidade de materiais (balança, fita, etc.)^11,16,99,129^; menor diversidade de cadernetas^22,130^.		
• Maior escolaridade da mãe/cuidador^17,59,63,96,132,138^ • Cuidador principal ser pais ou avós^59^ • Realização de pré-natal^59^ • Pré-natal no SUS^59^ • Mãe/cuidador realizar anotações na CC^35,60^	• Mais novas^35,63^ • Nascidas à termo^60^ • Maior número de consultas de puericultura^35^	• Atuação na APS (vc MAC)^33,90,113,129^ • Crianças acompanhadas por médicos generalista (vs pediatra)^63^ • Comunicação entre profissionais de saúde^90,107,122,127,137,139,140^ • Conhecimentos sobre a CC^11,21,35,53, 77-79,88,90,95,99,113,127,128,130–132,137,139,141^ • Orientar/dialogar com os cuidadores sobre a CC^53,59,60,63,91,112,132,138,139^
**Fatores relacionados ao preenchimento das medidas antropométricas na CC**		
• Maior escolaridade da mãe/cuidador (peso^84,88^) • Renda mais alta (peso^83,88^; gráfico de altura^83^ • Renda mais baixa (peso^7^) • Mais velha (o) (≥ 20 anos-peso^84,88^) • Mais nova (o) (< 35 anos-peso^53^; altura^53^) • Mãe primípara (peso^88^) • Mães que não trabalham fora (peso^7^)	• Mais velhas (peso^53,88^; gráfico de peso^65^; altura^53^) • Mais novas (peso^83^; gráfico da altura^83^) • Maior número de consultas de puericultura (peso^97^) • Puericultura ESF (vs tradicional, altura^53^)	Orientar/dialogar com os cuidadores sobre a CC^84,131^
**Farotes relacionados ao preenchimento dos marcos do DI**		
• Maior escolaridade da mãe/cuidador^7^ • Renda mais alta^24^ • Mãe primípara^7,65,88^ • Não residir na área de atuação do ACS^7^	• Sexo feminino^88^ • Mais velhas^7^ • Não anêmicas^24^	• Orientar/dialogar com os cuidadores sobre a CC^131^
**Fatores relacionados a leitura da CC pela mãe e a levar a CC nas consultas**		
LEITURA • Maior escolaridade da mãe/cuidador^53,34^ • Pele branca^34^ • Mãe não ser chefe de família^34^ • Realização de ≥ 6 consultas pré-natal^34^	LEITURA • Mais velhas^53^LEVAR • Mais novas^94^	LEVAR • Solicitar a CC durante as consultas^84,96^ • Realizar anotações na CC^84,96^ • Orientar/dialogar com os cuidadores sobre a CC^9^
**Familiares e cuidadores**	**Crianças**	**Profissionais de saúde**

ESF: estratégia saúde da família; CC: Caderneta da Criança; APS: atenção primária à saúde; MAC: serviços de média e alta complexidade; DI: desenvolvimento infantil; ACS: agente comunitário de saúde.

Os itens de acompanhamento com maior prevalência de preenchimento foram vacinação e medidas antropométricas. O preenchimento dos itens de vacinação variou entre 91,8% e 100%^17,59,60,63,80,82,84,94^. Preenchimento de peso da criança variou de 2% a 96,3%^16,17,21, 25,27,34,53,59,83,86,88,94,97,125^ e essas medidas foram transpostas ao gráfico entre 9% e 100% das vezes^21,22,34,63,65,84,88,92,94,97,137^. Prevalências menores foram encontradas para preenchimento da altura, variando de 19% a 66,2%^16,53,59,94,137^, sendo que o gráfico da altura esteve preenchido de 8,9% a 100% dos casos^22,84,92,94^. Ainda menores prevalências de preenchimento foram encontradas para o perímetro cefálico, variando de 9,7% a 43,1%^88,137^, e gráfico dessa medida (21,9% a 72,7%)^22,59,92^.

O preenchimento dos marcos do DI variou entre 0% e 72,7%^7,16,17,24,27,34,35,59,60,65,82,84,88,92,94,98,122, 136,137^. Piores prevalências ou ausência de registros foram atribuídos à suplementação de ferro e vitamina A, saúde auditiva, ocular e bucal^22,33,60,63,82,84,141^.

### Fatores Associados ao Uso da CC

Os fatores associados ao uso inadequado da CC passam pelo conhecimento deficiente dos profissionais de saúde sobre a CC^11,21,35,53,77^ e muitos estudos ressaltam a falta de capacitações sobre a CC direcionadas a esses profissionais^11,12,16,21,22,25,35,53,88,90,96,99,112,127,129^.

A CC é mais utilizada no atendimento ambulatorial, não sendo muito valorizada na área hospitalar^33,90,113,129^. Apesar de reconhecerem que a falta de registros e uso descontinuado da CC prejudicam o acompanhamento da saúde da criança^127,130,139^, os profissionais de saúde a entendem principalmente como instrumento de registro da vacinação e crescimento^80,90,96,107,127,129^. Em falas desses profissionais, a CC aparece também como algo que permite e norteia o acompanhamento infantil pelos diferentes serviços^90,99,129^ e estimula a comunicação com familiares^78,90,96,98,127^, porém esses usos não são observados na prática^18,60,90,93,96,107,127,129,132,140^. Os agentes comunitários de saúde (ACS) aparecem como ponto estratégico para o trabalho com a CC^7^, porém não se sentem valorizados pela população, por observarem resistência das mães quando solicitam a CC para consulta^94^. Estudo que buscou descrever as práticas de visita domiciliar do ACS segundo região do país e localização das UBS identificou que a região Nordeste teve maiores percentuais de verificação do “cartão de vacinação” (51%)^126^.

Fatores associados ao processo de trabalho, como falta da CC^21,53,88,96,112,127,129,137,140^, alta demanda de atendimento^90,91,99,113,127,129,137^ e outros também influenciam o seu preenchimento (Quadro). Entre os estados da macrorregião do Nordeste, 46,9% das crianças de 0 a 2 anos receberam a CC, variando de 36,8% (Sergipe) a 58,8% (Ceará)^62^. Apenas 50–55% das crianças recebem a CC na maternidade^21,25^.

Os profissionais de saúde alegam que há falta de interesse e pouco envolvimento dos familiares e cuidadores no uso da CC – pois eles não leem nem levam a CC às consultas^90,91,94,96,112,129,132,137,139,140^–, mas acreditam que a CC possibilita o entendimento das mães sobre a saúde dos filhos^127^. Ainda, referem que os familiares deveriam exigir o preenchimento da CC^90^, corresponsabilizando-se pelo seu uso^90,99^. Por outro lado, reconhecem que a não utilização da CC pelos cuidadores possa estar relacionada ao fato de não receberem orientações para isso^91,112,132,139^. A família ou cuidador que recebeu orientação sobre a CC variou entre 33% e 64,3%^33,35,60,63,84,94,129^.

Do outro lado, apesar de reconhecerem a CC como documento da criança e tecnologia de auxílio no cuidado com os filhos^33,96,104^, familiares e cuidadores sentem-se excluídos do processo, uma vez que não há diálogo sobre o instrumento nem estímulo a sua leitura e uso^96,140^. Apesar disso, entre 80,3% e 88% das mães referiram ter lido a CC^34,35,53^. Vale ressaltar que entre 18% e 26% das mães fazem anotações nas cadernetas de seus filhos^35,60,63,84^, sendo esse fator associado a melhores índices de preenchimento^35,60^.

A porcentagem de familiares e cuidadores que tinham e portavam a CC na ocasião do estudo variou entre 46,9% e 100%^7,21,22,24,25,27,36,53,59,83,84,86,94,97,125^. Já a porcentagem de cuidadores que relataram levar a CC às consultas variou entre 76% e 93,5%^35,84,94^. Porém, alguns estudos identificaram que 52% das mães foram orientadas a levar a CC nas consultas^94^, de 70% a 86,5% dos profissionais de saúde solicitaram a CC durante a consulta^21,25,35^, 49% dos profissionais realizaram anotações na CC durante a consulta^94^ e 25% dos familiares e cuidadores perceberam indiferença do médico em relação à CC^84^. Segundo familiares e cuidadores, não receber orientações dos profissionais de saúde em relação a CC^53,138^ e a dificuldade de compreender as informações ali contidas^96,132,138^ são fatores associados ao uso reduzido da CC por esses atores.

O Quadro representa os fatores relacionados ao uso e/ou preenchimento da CC.

### Estudos de Intervenção

Nos quatro estudos de intervenção identificados^93,94,112,122^ as estratégias para ampliar o uso da CC passam pelo acompanhamento coletivo utilizando a CC^93^, lembretes adicionados ao prontuário da criança para que o profissional preencha a CC^122^ e criação de espaços de diálogo sobre a CC^112^. Um dos estudos^94^ alerta que capacitações podem não ter o impacto esperado, sendo necessário planejar estratégias para o alcance do público-alvo e o envolvimento dos gestores. A falta de espaço adequado, suporte e organização, sobrecarga de trabalho e a falta de participação dos profissionais de saúde foram barreiras para essas ações^93,94,112,122^.

### Instrumento para a Coleta de Dados

Um estudo utilizou a curva de peso para idade a fim de avaliar o estado nutricional de crianças de 6 a 60 meses utilizando duas metodologias (critérios de Waterlow = 42,9% e gráfico da CC = 35,6%), concluindo que a prevalência de crianças com problemas nutricionais foi semelhante e alta^29^.

Seis estudos utilizaram o instrumento de vigilância do DI da CC^19,23,28,46,57,89^. Um trabalho avaliou o desenvolvimento neuropsicomotor de crianças de 0 a 18 meses^28^ utilizando os marcos da CC, evidenciando que 53% delas apresentaram todos os marcos esperados para a idade. Outro estudo^46^ avaliou a acurácia do instrumento CC em relação ao teste de triagem Bayley III. O instrumento de vigilância da CC apresentou resultados moderados de sensibilidade (57,1%), especificidade (69,4%), acurácia (64%), valor preditivo positivo (59,5%) e valor preditivo negativo (67,3%), deixando de identificar 43% das crianças em alerta ou com provável atraso do desenvolvimento. Dois estudos^19,89^encontraram concordância baixa entre os instrumentos de vigilância da CC e o da Atenção Integrada às Doenças Prevalentes na Infância (AIDPI) entre crianças de dois meses a dois anos: concordância de 0,34 (coeficiente Kappa = -0,12; p = 0,98)^19^ e 31,6% na CC e 34,1% na AIDPI com atraso no DI (coeficiente Kappa=0,27)^89^. Outro encontrou concordância baixa entre a *Alberta Infant Motor Scale* (AIMS) e a CC (coeficiente Kappa = 0,077 a 0,096; p = 0,000)^23^. Não foi encontrada concordância entre o instrumento da CC e a escala *Harris Infant Neuromotor Test* (HINT) (coeficiente Kappa = -0,01)^57^.

Os estudos que buscam comparar o instrumento de vigilância do DI da CC com outros instrumentos encontram algumas dificuldades: (i) a quantidade de marcos avaliados^46^ e os indicadores de alterações do DI são baseados em critérios distinto^57,89^; (ii) as formas de pontuação e classificação são diferentes, podendo impactar os resultados^46^; (iii) o instrumento da CC não menciona a necessidade de correção da idade cronológica para crianças nascidas prematuras^46^; (iv) não há definição do tempo que o avaliador deve esperar ou número de tentativas para a realização de um determinado marco^46^; (v) o instrumento da CC adianta marcos do DI que deveriam ser exigidos em faixas etárias mais avançadas, deixando dúvidas em relação à classificação da criança^46,89^; e (vi) explicação vaga e linguagem imprecisa na elaboração do instrumento da CC^46,128^.

## DISCUSSÃO

Até onde sabemos, esta é a primeira revisão de escopo que compila toda a produção científica publicada entre 1990 e julho de 2021 sobre a CC no Brasil. Um número expressivo de trabalhos foi identificado (n = 129) e quase metade utilizou a CC como fonte de dados, principalmente dados de vacinação. A outra metade toma a CC como objeto de estudo, citando-a nos resultados ou utilizando-a como instrumento para a coleta de dados. Nesse caso, os principais desfechos investigados foram o preenchimento e os fatores associados ao preenchimento da CC, nos quais foram identificadas fragilidades no preenchimento de todos os itens, com exceção da vacinação. Características contextuais referentes a crianças, familiares e cuidadores, bem como a profissionais, foram relacionadas ao uso e/ou preenchimento da CC.

Notou-se que a CC vem contribuindo para o estudo das coberturas vacinais^20,26, 31,37^ e, em menor amplitude, para o estudo do estado nutricional das crianças^16,21,23,24,32,51,54,57,61,66,68,71,85,100,101,103,105,109,118^. Ou seja, à medida que a CC é adequadamente preenchida, ampliam-se as possibilidades de sua utilização para a análise de diferentes desfechos em saúde. O uso de uma nomenclatura inadequada demonstra que os autores podem estar limitando a CC ao seu uso inicial^11,80,141^, de registro da vacinação, e essa visão parece se perpetuar e ser transmitida aos cuidadores, não favorecendo a mudança para uma visão ampliada da CC. A CC é, também, um instrumento destinado ao empoderamento das famílias para os cuidados com a criança, mas esses atores não têm sido suficientemente envolvidos no processo de cuidado. Apesar dessas barreiras, estudos brasileiros apontam associação positiva entre a utilização da CC e melhores desfechos, como estado nutricional da criança^86^ e DI^142^.

Quase metade dos estudos foram conduzidos na região Nordeste. Um estudo de abrangência nacional sobre visitas domiciliares identificou maiores percentuais de verificação do “cartão de vacinação” (51%) e pesagem das crianças (41,3%) nessa região^126^, o que pode indicar que atividades de acompanhamento da saúde das crianças são prioridade no âmbito da APS nordestina. A APS foi o cenário da maior parte dos estudos, em consonância ao seu papel de coordenadora do cuidado nas redes de atenção à saúde^143^. Consequentemente, as UBS foram os locais preferenciais para realização dos estudos, como alternativa viável e de menor custo para a realização de pesquisas sobre a saúde infantil. Vale destacar a utilização das campanhas de vacinação como alternativa para a realização de inquéritos sobre saúde, nutrição e DI no Brasil^146,148^. Porém, deu-se pouca ênfase ao papel de outros pontos de atenção^139^, como as maternidades, que são fundamentais na distribuição e preenchimento inicial da CC.

O interesse por estudar a utilização da CC parece ter se iniciado em 2005, coincidindo com o lançamento da CSC, quando o documento passou a ser usado como instrumento de vigilância e acompanhamento integral da saúde da criança de forma mais enfática. O principal desfecho investigado pelos estudos que utilizaram a CC como objeto de estudo ou citaram-na em seus resultados foi o seu preenchimento e fatores associados ao preenchimento. Trata-se, portanto, da obtenção de um diagnóstico sobre o uso dessa ferramenta, podendo auxiliar na definição de estratégias para melhorar o cenário atualmente restrito de sua utilização. Porém, fica clara a necessidade da inclusão da temática da CC nos processos de educação permanente para incorporação desse instrumento como orientador das práticas de saúde^11,21,35,53,77^. Ainda, a produção no campo das pesquisas de implementação é pequena e poucos estudos avançam na proposição de modelos de intervenção para solucionar o problema^93,94,112,122^.

A incorporação da vigilância do DI no processo de trabalho da APS utilizando a CC é mais um desafio^122,136^. A proposta de realização do exame físico da criança e identificação de fatores de risco ao DI (biológicos, sociais e ambientais), e a observação dos marcos do desenvolvimento tornam a CC uma ferramenta de apoio aos profissionais para a vigilância do DI^144^. Dessa forma, deve-se interpretar com cautela os estudos que se propuseram a validar os marcos do DI da CC como um instrumento de triagem, uma vez que o propósito do MS é a utilização desses marcos em conjunto com outras informações para identificar crianças com possíveis atrasos. Assim, mais estudos são necessários considerando todos os aspectos levantados na CC para a vigilância do DI e seu propósito de avaliação global do DI.

A amplitude da busca bibliográfica realizada e a inserção de literatura cinzenta são pontos positivos desta revisão. Por outro lado, a exclusão de resumos de congresso e trabalhos de conclusão de curso, bem como a consulta limitada às primeiras dez páginas do Google Scholar e a não disponibilização de relatórios sobre a CC oriundos de estudos financiados pelo MS, podem ser consideradas limitações.

A CC em sua multifuncionalidade pode ser uma ferramenta formativa e de apoio a profissionais que atuam na área de saúde da criança e de orientação aos cuidadores, assim como os dados gerados com o seu preenchimento podem contribuir para a elaboração de políticas públicas envolvendo a primeira infância. Apesar disso, os resultados desta revisão reforçam a necessidade de compreender melhor quais fatores afetam a distribuição da CC à população e como aprimorar esse processo. Apontam também para a necessidade de compreender o seu uso e preenchimento pelos diversos pontos de atenção e setores, nos diferentes contextos brasileiros. Fica clara a necessidade de estudos que avaliem modelos de formação dos profissionais de saúde para o uso da CC no contexto da educação permanente em saúde e de intervenções que incentivem o uso da CC pelos cuidadores. Dessa forma, pesquisadores, formuladores de políticas, gestores, profissionais de saúde e de outros setores devem buscar na CC uma forma de efetivamente sistematizar e monitorar o cuidado à saúde da criança, fortalecendo essa importante estratégia inserida nas políticas de promoção do crescimento e desenvolvimento saudáveis e redução da morbimortalidade infantil.

## Footnotes

a Disponível em: https://drive.google.com/file/d/13yrMzEmMu1HbepRUceaSrVTA8zGTmrQg/view?usp=sharing

b Disponível em: https://drive.google.com/file/d/1TJwAobpWAHqYlvkqVIBlwnWtrFUHei7r/view?usp=sharing

c Disponível em: https://drive.google.com/file/d/17XxDuTlsQ1ifkIDlNFsmtdvcRVDOUPFW/view?usp=sharing
